# Data on the uptake of reducible antigen-adjuvant conjugates by dendritic cells

**DOI:** 10.1016/j.dib.2019.103759

**Published:** 2019-03-06

**Authors:** Katrin Kramer, Sarah L. Young, Greg F. Walker

**Affiliations:** a School of Pharmacy, University of Otago, Dunedin, New Zealand; b Department of Pathology, University of Otago, Dunedin, New Zealand

## Abstract

This article contains the uptake data of two reducible antigen-adjuvant conjugates with different sensitivities to the extracellular and intracellular reductive environment. Using a linker with different redox sensitivity the adjuvant cytosine-phosphate-guanine (CpG) was conjugated to the fluorescently labeled model tumour antigen ovalbumin (OVA). The uptake of the conjugates by dendritic cells in a total splenocyte culture was determined using flow cytometry. The data presented in this paper supports the finding in the research article “Intracellular cleavable CpG oligodeoxynucleotide – antigen conjugate enhances anti-tumour immunity” (Kramer et al., 2016) [1].

**Table:** Specifications table

Subject area	*Immunology, Drug Delivery*
More specific subject area	*Immunotherapy*
Type of data	*Graph*
How data was acquired	*Flow cytometry; Gallios flow cytometer by Beckman Coulter*
Data format	*Analysed*
Experimental factors	*Fluorescently labeled antigen-adjuvant conjugates were incubated with splenocyte culture from C5*7BL*/6 mice*
Experimental features	*Splenocytes were labeled with antibodies and detected on flow cytometer*
Data source location	*Dunedin, New Zealand*
Data accessibility	*Data is within this article*
Related research article	*Kramer K, Shields NJ, Poppe V, Young SL, Walker GF. Intracellular Cleavable CpG Oligodeoxynucleotide-Antigen Conjugate Enhances Anti-tumour Immunity. Mol Ther. 2017 Jan;25(1):62–70.*

**Table:** 

**Value of the data** • This data shows that the more stable reducible linker (HYN-SS) has a significantly higher uptake into DCs compared to the conjugate linked with the more sensitive reducible linker (SS). • This in vitro uptake data correlates with the in vivo finding in Kramer et al., 2016 [1]. • This data suggests that in vitro uptake studies could be useful for researchers who are designing reducible antigen-adjuvant conjugate systems for delivery to antigen-presenting cells

## 1 Data

**Fig. 1:**
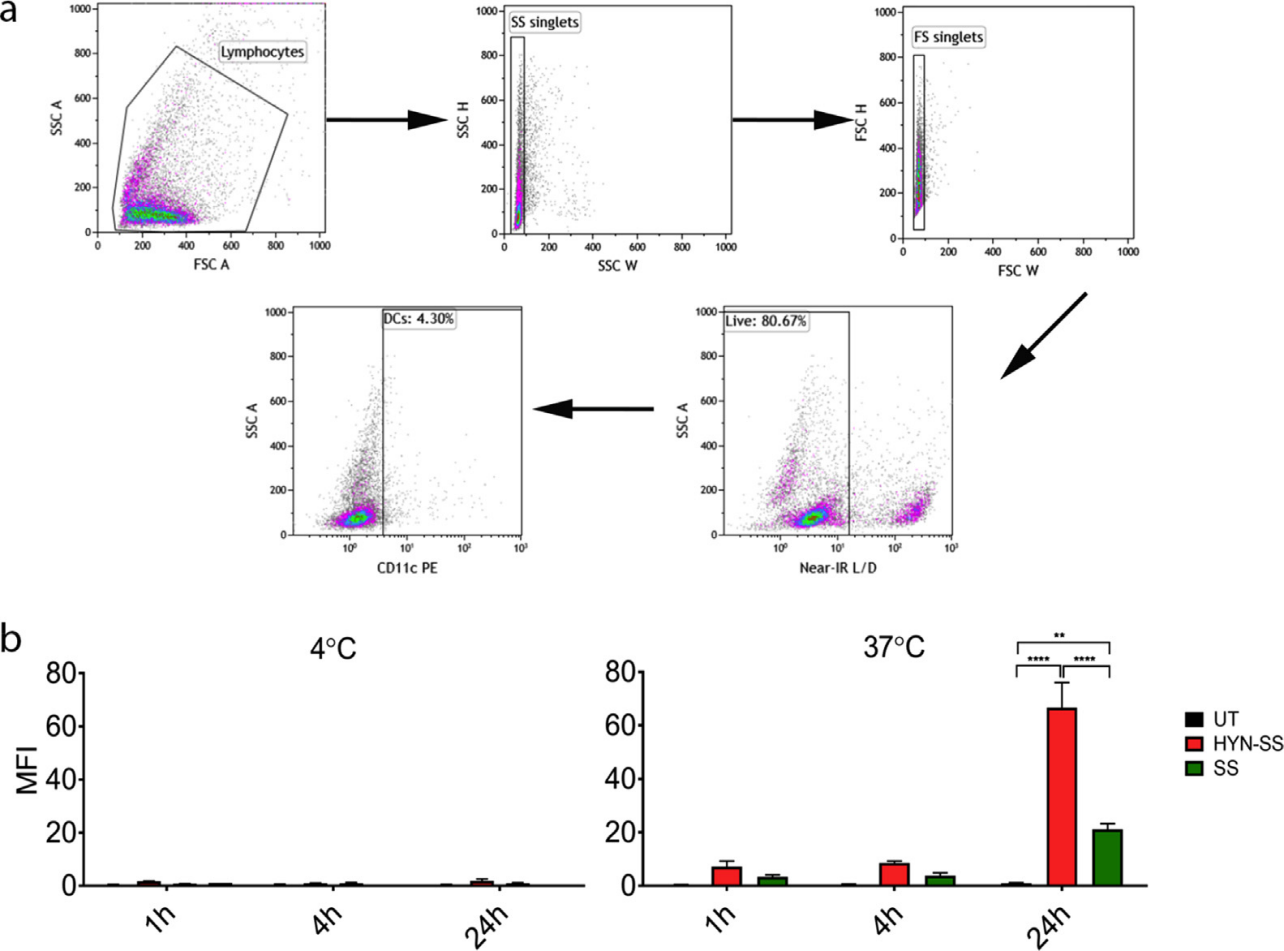
Uptake of the CpG-OVA conjugates HYN-SS and SS by splenocytes. Splenocytes were incubated at 4 °C and 37 °C with fluorescently labeled (DyLight633) conjugates or media as control. a) Following incubation for 1h, 4h and 24h splenocytes were gated on size and granularity, single cells, live cells and CD11c + dendritic cells. b) Uptake results are expressed as median fluorescent intensity (MFI) measured by flow cytometry of cells of interest. Bars represent the mean of three independent experiments ±SEM, statistical significance was determined by two-way ANOVA with Bonferroni's post-hoc test, ****p < 0.0001, ***p < 0.001; **p < 0.01, *p < 0.05. Statistical significance displayed is the comparison of conjugate treatment to treatment with mixture of CpG and OVA.

This data article refers to the research article “Intracellular cleavable CpG oligodeoxynucleotide - antigen conjugate enhances anti-tumour immunity” [1]. Conjugates of the vaccine adjuvant CpG and the model tumour antigen OVA were made as improved immunotherapeutic agents. Two conjugates were synthesised, an intracellular cleavable one (HYN-SS) and an extracellular cleavable one (SS). The data here presents uptake of the CpG-OVA conjugates by a dendritic cell population of murine splenocytes in vitro. To identify fluorescently labeled conjugate taken up by dendritic cells, splenocytes were gated on size and granularity, single cells, viability and CD11c + dendritic cells (Fig. 1a). The intracellular cleavable HYN-SS conjugate showed statistically higher uptake compared to the extracellular cleaved SS conjugate (Fig. 1b).

## 2 Experimental design, materials and methods

### 2.1 Synthesis of fluorescent conjugates

OVA (Sigma–Aldrich, Saint Louis, MO, USA) was reconstituted in PBS and purified from aggregates via size-exclusion chromatography (Superdex™ 200 10/300 GL; GE Healthcare Bio-Sciences, Uppsala, SE) using PBS as the elution buffer. Purified OVA monomer was added to lyophilised NHS-DyLight633 and incubated for 1 h at room temperature. The degree of DyLight633 labelling on OVA was calculated using the extinction coefficient of 170,000 M-1cm-1 for DyLight633.

Oligonucleotide CpG 1668 (5′-TCCATGACGTTCCTGATGCT-3′) with a phosphorothioate backbone modified with a 3′ amine (CpG-NH_2_) or 3′ thiol modification (CpG-SH) was obtained from GeneWorks Pty Ltd (Hindmarsh, SA, AUS).

For the HYN-SS conjugate, OVA-DyLight was modified with a 30-fold molar excess of succinimidyl 6-hydrazinonicotinate acetone hydrazone (HyNic from Solulink Inc., San Diego, CA, USA) in 0.1 M sodium phosphate, 0.15 M NaCl, pH 8 for 2 h at room temperature, and excess linker was removed by buffer exchange with PBS using a Vivaspin 2 spin filter. CpG-NH_2_ was modified with a 30-fold molar excess of succinimidyl—SS—4-formylbenzoate (4FB-SS from Solulink) for 2 h at room temperature. HyNic-modified OVA (DyLight labeled) was reacted with the modified CpG at a 1:4 M ratio for 2 h at room temperature to form a stable bis-arylhydrazone bond. The conjugates were purified by size-exclusion chromatography (Superdex™ 200 10/300 GL).

Protein concentration of the conjugate was measured by Quant-iT™ Protein Assay Kit (ThermoFisher Scientific).

For SS conjugate, succinimidyl 6-(3-[2-pyridyldithio]-propionamido) hexanoate (LC-SPDP, Thermo Fisher Scientific) linker was prepared by dissolving it at a concentration of 20 mM in dimethyl sulfoxide (DMSO). OVA (DyLight labeled) in PBS was added to the LC-SPDP linker at a 30-fold molar excess of the linker and incubated for 30 min at room temperature. CpG-SH was added to pyridyldithiol-activated OVA at a molar ratio of 4:1 overnight at room temperature. The SS conjugate was purified by size-exclusion chromatography (Superdex™ 200 10/300 GL) and protein concentration was measured by Quant-iT™ Protein Assay Kit (ThermoFisher Scientific).

### 2.2 Experimental mice

Female C57BL/6 mice aged 6–12 weeks were obtained from the Hercus Taieri Research Unit, University of Otago. Experiments were conducted in accordance with ethical permits granted by the University of Otago Animal Ethics Committee (AEC 09/14). All animals were euthanized by cervical dislocation.

### 2.3 Immunofluorescent analysis of conjugate internalisation

Splenocytes from C57BL/6 mice were prepared as a single cell suspension and treated with ammonium chloride to lyse red blood cells. Cells were resuspended at 1 × 10^6^ cells/ml in cIMDM+5%FCS, plated with 100 μl per well and either pre-cooled to 4 °C or pre-warmed to 37 °C for 30 min. The cells were pulsed with 7 μg/ml of DyLight633 labeled conjugate (HYN-SS or SS). Following incubation at 37 °C, 5% CO2 or 4 °C for 1, 4 or 24 h, the cells were harvested and stained with Live Dead near IR, treated with Fc block and stained with antibodies to identify DCs (APC anti-CD11c). Fluorescence was measured using a Gallios flow cytometer and analysed using Kaluza software version. Statistical analysis of uptake was performed using GraphPad Prism version 6.0b. Statistical analysis was carried out using one-way analysis of variance (ANOVA) with Dunnett's post-hoc test to compare the difference of one variation in more than two different treatment groups, two-way ANOVA with Bonferroni's post-hoc test to compare the difference of two variations in different treatment groups. The particular type of statistical analysis is indicated in each relevant figure legend. Error bars in the graphs represent standard error of the mean (SEM).
